# Efficacy and safety of cryotherapy, cold cone or thermocoagulation compared to LEEP as a therapy for cervical intraepithelial neoplasia: Systematic review.

**DOI:** 10.11606/s1518-8787.2020054001750

**Published:** 2020-03-09

**Authors:** Yamilée Hurtado-Roca, Naysha Becerra-Chauca, Magaly Malca

**Affiliations:** I Instituto de Evaluación de Tecnologías en Salud e InvestigaciónEsSaludLimaPerú Instituto de Evaluación de Tecnologías en Salud e Investigación (IETSI), EsSalud . Lima , Perú; II Hospital Rebagliati MartinsServicio de Ginecología OncológicaEsSaludLimaPerú Hospital Rebagliati Martins , Servicio de Ginecología Oncológica , EsSalud . Lima , Perú

**Keywords:** Cervical Intraepithelial Neoplasia, therapy, Cryotherapy, Conization, Electrosurgery, Uterine Cervical Neoplasms, Systematic Review

## Abstract

**OBJECTIVES:**

To determine the efficacy and safety of the use of cryotherapy, cold knife or thermocoagulation compared to Loop Electrosurgical Excision Procedure (LEEP) for the treatment of cervical intraepithelial neoplasia.

**METHODS:**

Systematic review with meta-analysis of randomized controlled trials in women with cervical intraepithelial neoplasia undergoing treatment with cryotherapy, cold knife, or thermo-coagulation compared with LEEP, to estimate its efficacy and safety. The search was conducted on MEDLINE/PUBMED, Cochrane Central Register of Controlled Trials (CENTRAL) and Scopus, until September 2018.

**RESULTS:**

The total of 72 studies were identified, of which only 8 studies met the inclusion criteria. The treatment of CIN with cold knife decreases the risk of residual disease compared with LEEP (RR, 0.54, 95%CI, 0.30–0.96, p = 0.04). The management of premalignant lesions with cryotherapy, compared with LEEP, increases the risk of disease recurrence by 86% (RR, 1.86, 95%CI, 1.16–2.97, p = 0.01), increases the risk of infections (RR, 1.17, 95%CI, 1.08–1.28, p < 0.001) and reduces the risk of minor bleeding by 51% (RR, 0.49, 95%CI) %, 0.40–0.59, p ≤ 0.001).

**CONCLUSIONS:**

The treatment of premalignant lesions of cervical cancer with cold knife reduces the risk of residual disease. Nevertheless, cryotherapy reduces the risk of minor bleeding in the 24 hours after treatment and increases the risk of recurrence of disease and infections.

## INTRODUCTION

Cervical cancer is the fourth most common type of cancer and the fourth leading cause of death in women worldwide ^1^ . However, cervical cancer still ranks as the second leading cause of death and the second most common cancer in the female population in low and middle income countries ^1^ . Cervical intraepithelial neoplasia (CIN) is a premalignant lesion of cervical cancer, histologically divided as CIN1, CIN2 and CIN3. Both these premalignant lesions and cancer *in situ* are attributed to human papillomavirus (HPV) ^2 , 3^ . According to the International Agency for Research on Cancer (IARC), report of 2018 ^4^ , in Peru, oncogenic HPV types 16 and 18 are found in 6.6% of patients with normal cytology, 27.3% with CIN1, 53.1% with CIN2-3 and 65.9% with cancer *in situ* . Early diagnosis and management of these pre-malignant lesions helps reduce the natural progression of these lesions into cervical uterine cancer. Monitoring of CIN2 and CIN3 histological lesions becomes a fundamental task in public health given that 31.0% of these evolve into cancer in the following 30 years ^5^ . Timely and appropriate therapeutic intervention can reduce this risk. Some authors have shown in monitoring cohorts of 10–20 years that the post-treatment rate of premalignant lesions decreases more than 30.0% during the first 10 years ^6 , 7^ .

For the treatment of premalignant cervical lesions, both ablative methods (cervical cryotherapy, laser ablation) and excisional methods (Loop Electrosurgical Excision Procedure (LEEP), cold cone) can be effective. As recommended by the World Health Organization guidelines in the clinical guide published in 2015 ^8^ , cryotherapy treatment is recommended for patients with CIN2+ lesions. If the patient is not eligible for this ablative therapy, the use of LEEP is recommended. However, excision and ablation procedures may be associated with adverse outcomes. Therefore, the objective of this systematic review and meta-analysis was to assess the effectiveness and safety of treating CIN with cryotherapy, cold cone or thermocoagulation compared to LEEP.

## METHODS

PRISMA (Preferred Reporting Items for Systematic Reviews and Meta-Analyses) guidelines were applied in the development and reporting of this systematic review ^9^ .

Randomized controlled trials (RCT) were considered eligible (as the objective was to assess efficacy and safety) that met the following criteria: study population women over 18 years old with diagnosed cervical intraepithelial neoplasia and treatment interventions with cryotherapy, cold cone, or thermocoagulation and LEEP as comparator. Studies were excluded if participants were pregnant women, women with HIV (Human Immunodeficiency Virus) infection, women with symptoms or a history of treatment and monitoring of cervical cancer.

The variables to assess effectiveness were: residual disease (in less than six months), recurrent disease (in more than six months) and positive margins. Major bleeding (hospitalization or blood transfusion), minor bleeding (bleeding not requiring hospitalization or blood transfusion after 24 hours after treatment), mortality associated with treatment, cervical stenosis, pain in treatment area, infections related to the procedure (requiring hospitalization or antibiotics) and damage to other organs or need for other surgeries were assessed.

We reviewed the MEDLINE/PUBMED, Cochrane Central Register of Controlled Trials (CENTRAL) The Cochrane Library and Scopus databases, using “Medical Subject Headings (MeSH)” or equivalent terms and text word terms. Articles in English and Spanish were included. A preliminary search strategy was created for MEDLINE/PUBMED. The remaining searches were tailored to individual databases ( Table 1 ), and the search was conducted from January 1993 to September 2018 (last 25 years). Additionally, we assessed the bibliographic references of the selected articles to identify other articles related to our systematic review. We used a broad search strategy aiming to increase sensitivity and identifying a relevant number of articles related to our research question. We developed the following MEDLINE search strategy:

**Table 1:** Characteristics of the studies included (1993–2018).

Study	Intervention			Histology	Study design	Study Period	Country	Monitoring Time	Type of study center	Professional who did the procedure	Outcome
	Cold Cone	Cryotherapy	LEEP								
Chirenje 2001	-	200	200	NIC 2-3	RCT	1997–1998	Zimbabwe	12 months	Screening Center	Gynecologist	Disease recurrence (after 6 months of treatment) and Residual disease (within 6 months of treatment)
Mitchell 1998	-	139	130	NIC 1-3	RCT	1992–1994	EUA	24 months	Specialized center	Three gynecologists, two family doctors and two nurse practitioners	Recurrence of illness (after 6 months of treatment), residual illness (within 6 months of treatment), cervical stenosis (requiring dilation), minor bleeding (requiring hospital visit), secondary pain (requiring medication) and infections (requiring antibiotic treatment).
Duggan 1999	89	-	91	NIC 1-3	RCT	1992–1994	EUA	12 months	Specialized women's hospital	Gynecology residents, with direct supervision by the researcher	Recurrence of illness (NS), Residual illness (NS), Minor bleeding (up to 6 weeks post-op), Cervical stenosis (NS), Infections (NS).
Giacalone 1999	38	-	28	NIC 2-3	RCT	1997–1998	France	3 months	University Hospital	Trained physician	Residual disease (NS), Secondary bleeding (requiring hospital visit), positive margins and cervical stenosis (inability to insert Heger dilator Nº3).
Girardi 1994	38	-	52	NIC 1-3	RCT	unspecified	Austria	10 months	University	Not specified	Recurrence of illness (up to 8 or 10 months), minor bleeding (NS)
Mathevet 1994	37	-	36	NIC 1-3	RCT	1990–1992	Canada	6 months	General Hospital	Trained physicians	Residual disease (NS), cervical stenosis (inability to insert a Heger Nº3 dilator), minor bleeding (NS) and positive margins.
Mathevet 2003	37	-	36	NIC 1-3	RCT	1990–1992	Canada	65 months	General Hospital	Trained physicians	Residual disease, cervical stenosis, minor bleeding and positive margins.
Takac 1999	120	-	120	NIC 1-3	RCT	1993–1996	Slovenia	3 months	Specialized hospital	Not specified	Residual disease (up to 3 months), minor bleeding (NS).

LEEP (Loop Electrosurgical Excision Procedure)

RCT: Randomized clinical trial. CIN: Cervical intraepithelial neoplasia.

NS: Not Specified

#1 (“Uterine Cervical Neoplasms”[Mesh]) OR ((“Uterine Cervical”[tiab] OR cervix[tiab]) AND (cancer[tiab] OR tumor[tiab] OR neoplasm[tiab] OR carcinoma[tiab] OR malignancy[tiab]))

#2 “Loop Electrosurgical Excision Procedure”[tiab] OR “Large loop excision of the transformation zone”[tiab] OR “LEEP”[tiab] OR “LLETZ”[tiab]

#3 “cryotherapy”[MeSH Terms] OR cryotherapy[tiab]

#4 “cold coagulation”[tiab]

#5 “conization”[MeSH Terms] OR “conization”[tiab]

#6 “Randomized Controlled Trial” [pt] OR “Randomized Controlled Trial”[tiab]

#7 #3 OR #4 OR #5

#8 AND #2 AND #7

#9 #8 AND #6

For the databases Scopus, and The Cochrane Library the search strategy was developed with the terms: “Uterine Cervical Neoplasms,” “Loop Electrosurgical Excision Procedure” and “Randomized Controlled Trial.”

### Study selection and data collection

Two reviewers independently assessed study eligibility based on titles and abstracts (YHR and NBC); discrepancies were solved by a third reviewer (JG). Titles and abstracts of all selected references were independently assessed by applying the objectives and research question (PICO). We manually reviewed all references in the selected full texts.

According to the search criteria in the different pre-selected databases, the references of each database that met the search criteria were exported to the Zotero software. Duplicates were eliminated and the required information (description of the methodology, results and conclusions) was extracted independently. Review Manager 5.3 (RevMan 5.3, Copenhagen, The Cochrane Collaboration) was used to extract the main data ^10^ .

To assess the risk of bias of each of the studies, we used the tool proposed by Higgins ^11^ , following the methodology of the Cochrane Collaboration. This assessment included the domains: sequence generation, assignation concealment, blinding, incomplete outcome data, and selective outcome reporting and other sources of bias.

The GRADE (Grading of Recommendations Assessment, Development and Evaluation) ^12^ methodology was used to assess the certainty of the evidence for each outcome. This assessment included the risk of bias, inconsistency, imprecision, indirect evidence and other considerations ^13^ .

Quantitative synthesis of eligible studies for binary outcomes was performed using a fixed effects model allowing the estimation of pooled RR and 95% confidence intervals of the effect of treatments for CIN from similar studies. Random effects sensitivity analyses were performed if the results were heterogeneous (I ^2^ > 50%). Heterogeneity tests (using the chi-square test of heterogeneity and the I ^2^ statistical test) were performed for each of the outcomes. Heterogeneity was accepted if I ^2^ > 50% and 0.1 p was selected as the cutting point for rejecting the null hypothesis of the study’s homogeneity ^14^ .

This study did not require ethics committee approval because it is not an identifiable data or individual data.

## RESULTS

**Figure 1:**
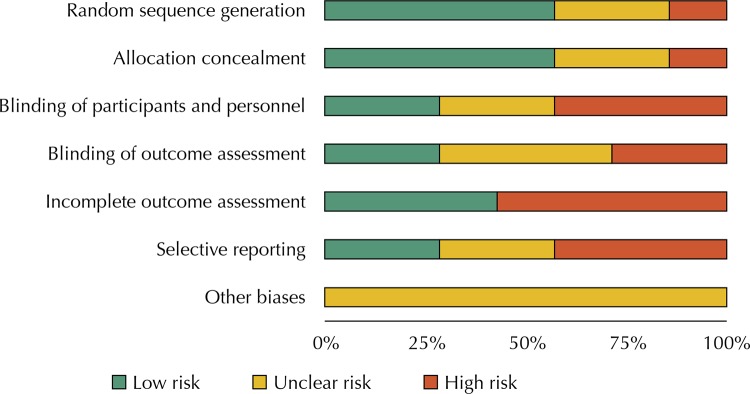
Domain Summary of Bias Risk Assessment according to the Cochrane Tool (1993–2018).

Seventy-two scientific articles were identified: 66 of the records identified through database searching (Cochrane = 22, PubMed = 19 and Scopus = 25) and six using additional records identified by other sources. After eliminating duplicates, 51 records were left for abstract reading, with 42 articles eliminated. After applying the selection criteria and after reading the full text, eight original articles were selected ^15^ ( Table 1 ). The article by Huang et al. ^22^ was excluded because it was not a randomized controlled study. Of the studies included, 57.0% had adequate randomized generation of the sequence and clearly described the method of allocation concealment; 29.0% carried out a blinding of participants and staff and blinding of the assessment of the event. Four studies had incomplete data to assess at the end of treatment which would generate a risk of bias due to loss to follow-up. Three of the studies did not clearly specify the outcomes to be assessed, so this was considered a high risk of reporting bias ( Figure 1 ). A better rating of the assessment of risk of bias could be seen in the studies by Duggan et al. ^19^ , Giacalone et al. ^15^ , and Mitchell et al. ^17^ .

Six of the included studies assessed treatment of CIN with cold cone compared with LEEP ^15 , 16 , 19^ , two studies assessed treatment with cryotherapy compared to LEEP ^17 , 18^ . No studies were identified assessing treatment of CIN with thermocoagulation compared with LEEP. Regarding the comparison between Cold Cone versus LEEP, the certainty of the evidence was very low for all outcomes ( Table 2 ). For the comparison between cryotherapy and LEEP, with the exception of the Minor Bleeding outcome after the first 24 hours post treatment which was of moderate certainty, the other outcomes were of low or very low certainty ( Table 2 ).

**Table 2:** Evidence Summary GRADE (1993–2018)

Outcome	Absolute effects Anticipated		Relative Effect (95%CI)	Nº of participants (Studies)	Quality of Evidence (GRADE)
	LEEP risk	Cold cone risk			
Question: Should Cold Cone vs. LEEP be used for treatment of Cervical Intraepithelial Neoplasia (CIN)? Bibliography: Girardi et al. 1994; Mathevet et al., 1994; Duggan et al., 1999; Takac et al. 1999; Giacalone et al., 1999; Mathevet et al., 2003					
Disease Recurrence (monitoring: range 6 months to 118 months)	71 per 1,000	23 per 1,000 (6 to 81)	RR 0.32 (0.09 to 1.14)	287 (3 ECAs)	⨁◯◯◯ VERY LOW ^a,b^
Residual Disease: (monitoring: up to 6 months)	112 per 1,000	61 per 1,000 (34 to 108)	RR 0.54 (0.30 to 0.96)	529 (4 ECAs)	⨁◯◯◯ VERY LOW ^c,d^
Positive Margins	212 per 1,000	164 per 1,000 (115 to 232)	RR 0.77 (0.54 to 1.09)	553 (4 ECAs)	⨁◯◯◯ VERY LOW ^c,d,e^
Minor bleeding during the first 24 hours after treatment	54 per 1,000	57 per 1,000 (27 to 119)	RR 1.05 (0.50 to 2.21)	469 (4 ECAs)	⨁◯◯◯ VERY LOW ^c,f^
Minor bleeding after the first 24 hours after treatment	88 per 1,000	83 per 1,000 (36 to 187)	RR 0.94 (0.41 to 0.13)	247 (2 ECAs)	⨁◯◯◯ VERY LOW ^c,f^
Cervical Stenosis (monitoring: range 3 months to 24 months)	66 per 1,000	70 per 1,000 (29 to 169)	RR 1.06 (0.44 to 2.58)	529 (4 ECAs)	⨁◯◯◯ VERY LOW ^c,f^

**Outcome**	**Absolute effects Anticipated**		**Relative Effect (95%CI)**	**Nº of participants (Studies)**	**Quality of Evidence (GRADE)**
	**LEEP risk**	**Cryotherapy Risk**			
Question: Should Cryotherapy vs. LEEP be used for treatment of Cervical Intraepithelial Neoplasia (CIN)? Bibliography: Mitchell et al., 1998; Chirenje et al., 2001					
Disease Recurrence (monitoring: range 6 to 24 months)	77 per 1,000	144 per 1,000 (90 to 229)	RR 1.86 (1.16 to 2.97)	598 (2 ECAs)	⨁◯◯◯ VERY LOW ^a,b^
Residual Disease: (monitoring: up to 6 months)	37 per 1,000	65 per 1,000 (31 to 133)	RR 1.75 (0.85 to 3.60)	596 (2 ECAs)	⨁◯◯◯ VERY LOW ^a,b^
Minor bleeding during the first 24 hours after treatment	15 per 1,000	4 per 1,000 (1 to 25)	RR 0.27 (0.04 to 1.62)	669 (2 ECAs)	⨁◯◯◯ VERY LOW ^c,d^
Minor bleeding after the first 24 hours after treatment	484 per 1,000	237 per 1,000 (194 to 286)	RR 0.49 (0.40 to 0.59)	625 (2 ECAs)	⨁⨁⨁◯ MODERATE ^c^
Cervical Stenosis: (monitoring: up to 24 months)	3 per 1,000	6 per 1,000 (1 to 68)	RR 1.87 (0.17 to 20.38)	596 (2 ECAs)	⨁◯◯◯ VERY LOW ^a,d^
Pain after 24h post treatment	275 per 1,000	256 per 1,000 (204 to 322)	RR 0.93 (0.74 to 1.17)	625 (2 ECAs)	⨁⨁◯◯ LOW ^e,f^
Infecciones después de 24h post tratamiento	465 per 1,000	544 per 1,000 (502 to 595)	RR 1.17 (1.08 to 1.28)	625 (2 ECAs)	⨁◯◯◯ VERY LOW ^a,b^

GRADE (Degrees of Certainty of Evidence); LEEP: Loop Electrosurgical Excision Procedure

High certainty: we are very sure that the real effect is similar to the estimation of the effect

Moderate certainty: we are moderately confident in the estimate of the effect: the actual effect is likely to be close to the estimate of the effect, but there is a possibility that it will be substantially different

Low certainty: our confidence in the estimate of the effect is limited: the actual effect may be substantially different from the estimate of the effect

Very low certainty: we have very little confidence in the estimate of the effect: the actual effect is likely to be substantially different from the estimate of the effect

a. It was decided to decrease two levels due to High Risk of Wearing Bias (both studies had more than 10% losses)

b. It was decided to decrease one level in imprecision because of the wide CI crossing the 1.25 limit.

c. It was decided to decrease one level for Uncertainty Risk of detection bias (uncertainty in blinding of assessors)

d. It was decided to decrease two levels in inaccuracy because of the wide CI crossing the limits of 0.75 and 1.25 and because of the small number of events.

e. It was decided to decrease one level for Uncertainty Risk of detection (blinding of assessors).

f. It was decided to decrease one level in imprecision because of the wide CI that crosses the 0.75 limit.

**Figure 2:**
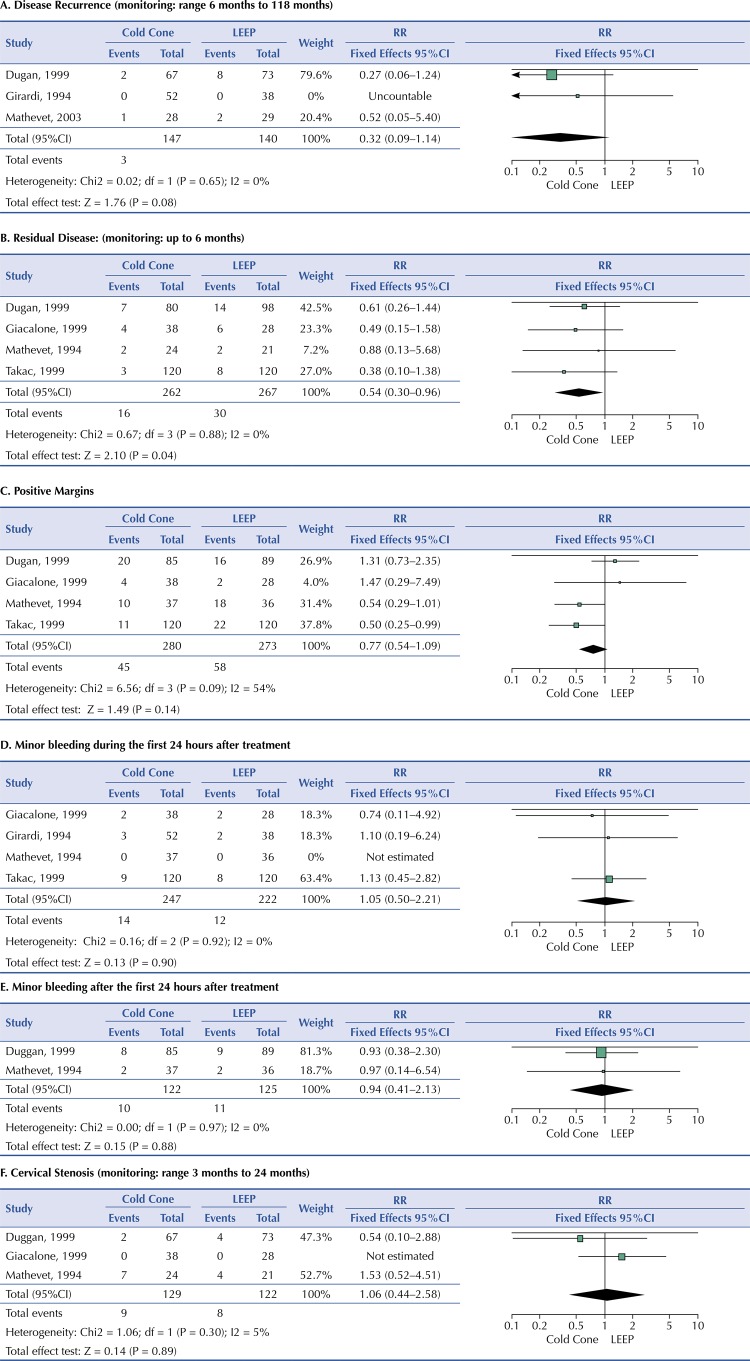
Treatment of cervical intraepithelial neoplasia (CIN) with cold cone versus Loop Electrosurgical Excision Procedure (LEEP).

Three of the six included studies assessed disease recurrence ^19 , 20 , 23^ . The prevalence of disease recurrence after cold cone treatment was 2.0% and in patients treated with LEEP, 7.1%. The study by Girardi et al. ^20^ did not identify any cases of disease recurrence in any of the patients treated with cold cone or LEEP. Meta-analysis of the studies showed no statistically significant difference in risk of disease recurrence between patients treated for CIN with cold cone compared to LEEP (RR 0.32, 95%CI, 0.09–1.14, p = 0.08). The studies showed no significant heterogeneity (P = 0.65 and I ^2^ = 0%) (Figure 2A).

Four studies assessed residual disease ^15 , 16 , 19 , 21^ . Residual disease was reported in 6.1% of patients treated with cold cone and in 11.2% of those treated with LEEP. Meta-analysis of all four studies showed that patients treated with cold cone were less likely to have residual disease compared to those treated with LEEP (RR 0.54, 95%CI, 0.30–0.96, p = 0.04). The studies showed no significant heterogeneity (P = 0.88 and I ^2^ = 0%) (Figure 2B).

Four studies assessed positive margins ^15 , 16 , 20 , 21^ . Cases were reported with both cold cone and LEEP use. In patients treated with cold cone, 16.1% of cases were reported and in those treated with LEEP 21.3% of cases were recorded. The meta-analysis of the four studies showed that there is no statistically significant risk of positive margins in patients treated for CIN with cold cone compared to LEEP (RR 0.77, 95%CI, 0.54–1.09, p = 0.14) (Figure 2C). The studies showed moderate heterogeneity (P = 0.09 and I ^2^ = 54%), so a sensitivity analysis using random effects was performed, and there was no significant change in result (RR 0.77, 95%CI, 0.44–1.35, p = 0.36).

Four included studies assessed this outcome ^15 , 16 , 20 , 21^ . The prevalence of minor bleeding in patients treated with cold cone was 5.7% and in those treated with LEEP 5.4%. After meta-analysis of the five studies, the risk of minor bleeding during the first 24 hours after treatment in patients treated with cold cone versus LEEP was not statistically significant (RR 1.05, 95%CI, 0.50–2.21, p = 0.90). No significant heterogeneity of the included studies was identified (P = 0.92 and I ^2^ = 0%) (Figure 2D).

Two studies assessed minor bleeding after the first 24 post-treatment ^16 , 19^ . 8.2% of patients treated with cold cone reported minor bleeding events after 24 hours of treatment, the prevalence of this event in patients treated with LEEP was 8.8%. After meta-analysis of the studies, the risk of minor bleeding after the first 24 hours after treatment in patients treated with cold cone versus LEEP was not statistically significant (RR, 0.94, 95%CI, 0.41–2.13, p = 0.88). No significant heterogeneity of the included studies was identified (P = 0.97 and I ^2^ = 0%) (Figure 2E).

Three studies assessed cervical stenosis ^15 , 16 , 19^ . The prevalence after cold cone treatment was 6.9% and 6.5% in patients treated with LEEP. The meta-analysis showed an increased risk of cervical stenosis in women treated with cold cone versus LEEP, however this result was not statistically significant (RR 1.06, 95%CI 0.44–2.58, p = 0.89). Giacalone et al. ^15^ did not identify cases of cervical stenosis in any of the patients treated with cold cone or LEEP. The studies showed low heterogeneity (P = 0.30 and I ^2^ = 5%) (Figure 2F).

Dugan et al. ^19^ reported one case of infection at the location of surgery in each group. The six studies assessed reported no major bleeding or pain secondary to cold cone treatment or LEEP.

**Figure 3:**
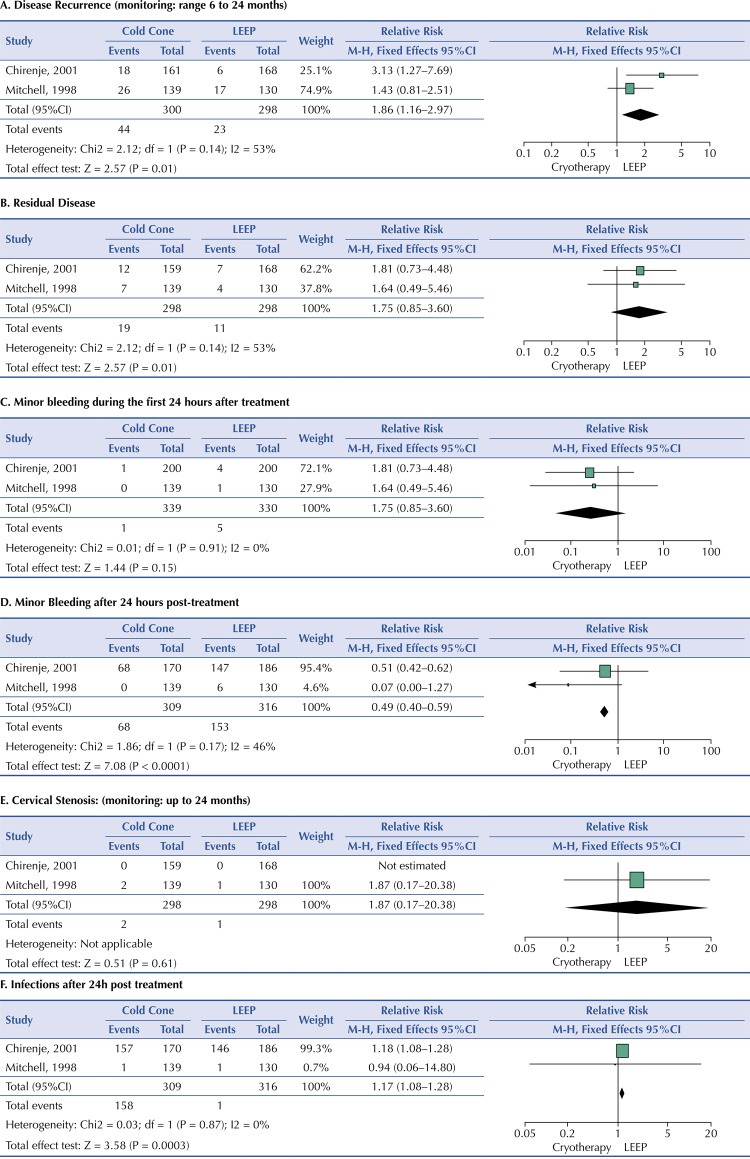
Treatment of cervical intraepithelial neoplasia (CIN) with cryotherapy versus Loop Electrosurgical Excision Procedure (LEEP).

For the comparison of CIN treatments with Cryotherapy versus LEEP, we identified two studies ^17 , 18^ . Both studies reported 14.7% disease recurrence in patients treated with cryotherapy and 7.7% of those treated with LEEP. The meta-analysis showed an increased risk of disease recurrence in women treated with cryotherapy compared to those treated with LEEP (RR 1.86, 95%CI, 1.16–2.97, p = 0.01) (Figure 3A). The studies showed moderate heterogeneity (P = 0.14 and I ^2^ = 53%) so a sensitivity analysis using random effects was performed, and there was no significant change in outcome (RR 1.86, 95%CI, 1.16–2.97, p = 0.01).

For treatment with cryotherapy versus LEEP, cases of residual disease were reported in the two selected studies. 6.3% of patients treated with cryotherapy and 3.7% of those treated with LEEP had this event. The meta-analysis showed an increased risk of residual disease in women treated with cryotherapy compared to those treated with LEEP. However, it was not statistically significant (RR 1.75, 95%CI, 0.85–3.60, p = 0.13). The studies showed no heterogeneity (P = 0.91 and I ^2^ = 0%) (Figure 3B). The prevalence of minor bleeding in patients treated with cryotherapy was 0. 3% and 1.5% in those treated with LEEP. After conducting the meta-analysis of the studies, the risk of minor bleeding during the first 24 hours after treatment in cryotherapy versus LEEP patients was not statistically significant (RR 0.27, 95%CI, 0.04–1.62, p = 0.15). No significant heterogeneity was identified from the included studies (P = 0.91 and I ^2^ = 0%) (Figure 3C).

22.0% of patients treated with cryotherapy reported minor bleeding events within 24 hours of treatment; the prevalence of this event in patients treated with LEEP was 48.4%. After conducting the meta-analysis, cryotherapy patients had a lower risk of bleeding after 24 hours of treatment compared to LEEP patients (RR 0.49, 95%CI, 0.40–0.59, p < 0.001). No significant heterogeneity was identified from the included studies (P = 0.17 and I ^2^ = 46%) (Figure 3D).

Chirenje et al. ^18^ did not report cases of cervical stenosis in any of the treatment groups. However, Mitchell et al. ^17^ reported two cases in the cryotherapy treated patients (139) and one case in the group of patients treated with LEEP (130), with a nonsignificant risk of cervical stenosis if treated with cryotherapy compared to LEEP (RR 1.87, 95%CI, 0.17–20.38, p = 0.61) (Figure 3E).

The two studies identified reported cases of pain secondary to treatment. The prevalence of pain in patients treated with cryotherapy was 23.9 percent and 27.5 percent in those treated with LEEP. The meta-analysis did not show a significant risk of pain secondary to cryotherapy treatment compared to LEEP (RR 0.93, 95%CI, 0.74–1.17, p = 0.54). The studies showed no heterogeneity (P = 0.50 and I ^2^ = 0%).

Both studies reported cases of post-treatment infections. The prevalence in the cryotherapy group was 51.1% and in the LEEP group was 46.5%. After meta-analysis of the studies, cryotherapy-treated patients were at increased risk for surgical wound infections compared to patients treated with LEEP (RR 1.17, 95%CI, 1.08–1.28, p < 0.001). The studies showed no heterogeneity (P = 0.87 and I ^2^ = 0%) (Figure 3F).

## DISCUSSION

In this systematic review, we identified few randomized controlled studies that assessed treatment of CIN with cryotherapy or cold cone compared to LEEP (two and six studies, respectively). The studies were of moderate methodological quality. No significant heterogeneity was identified among the studies assessed for each of the efficacy and safety outcomes, except for positive margins in the studies comparing cold cone versus LEEP and for disease recurrence for the studies comparing cryotherapy to LEEP. Meta-analysis of data from the included studies showed that cold cone use decreases the risk of residual disease compared to LEEP. While the use of cryotherapy increases the risk of disease recurrence and infection; however, it reduces the risk of minor bleeding compared to LEEP treatment. In contrast to the study by Jiang et al. ^24^ , we found that there is a lower risk of residual disease in patients treated with cold cone compared to LEEP. This finding may suggest that the technique used could be related to the depth of the extracted tissue, revealed in the positivity of the margins, which Jin et al. ^25^ in their meta-analysis, point out as a prognostic factor for recurrence and/or residual disease. The systematic review of El-Nashar et al. ^26^ showed a lower probability of postoperative bleeding and cervical stenosis in the management of CIN with LEEP. Their results show an increase of up to two times in the incidence of disease recurrence in patients treated with LEEP compared to those who were treated with cold cone. These results are at odds with our findings. We have not identified any differences in the management of premalignant lesions with these two methods for the same outcomes. This discrepancy can be attributed to the difference in the types of studies included in both reviews. The study by El-Nashar et al. ^26^ included both randomized controlled trials and observational studies and did not perform stratified analyses for each of these types of studies. Since the aim of our study was to assess efficacy and safety, our systematic review was restricted to the assessment of RCT.

Our study assessed the effectiveness and safety of managing CIN with cryotherapy compared to LEEP. A previous systematic review of controlled studies suggests that the rate of disease recurrence (≥ 6 months post-treatment) may be lower after management of CIN with LEEP compared to cryotherapy (RR 0.32, 95%CI 0.13–0.78) ^27^ . Our review also includes the study by Mitchell et al. ^17^ finding a nearly doubled rate of disease recurrence with the use of cryotherapy compared to LEEP. Therefore, our study is consistent with the evidence of increased risk of disease recurrence with the use of cryotherapy compared to LEEP. Similarly, D’Alessandro et al. ^28^ have published a systematic review assessing the management of CIN with LEEP and cryotherapy. The results of this study show a lower risk of disease recurrence in patients treated with LEEP compared to those treated with cryotherapy (RR 0.87; 95%CI, 0.76–0.99). This finding is similar to our review where we found the effectiveness of LEEP use to be greater relative to cryotherapy, as we identified an increased risk of disease recurrence with the use of cryotherapy. Our study did not include Smith et al. ^29^ because they assessed patients with HIV, as well as the Singh et al. ^30^ study because it was an observational study. It is essential to note the context in which the two included studies conducted the treatments: the professionals were health care workers specially trained in the use of both methods, and the care centers were specialized units that could increase the chances of success of the procedures. These are important points to consider when planning their implementation. The use of cold cone would be the best option compared to LEEP in patients with CIN, in terms of residual disease. On the other hand, the use of LEEP would be the best option compared to cryotherapy in terms of recurrence and post-operative infection. However, this evidence is of very low certainty, according to GRADE methodology. The true effect may be substantially different. More randomized controlled studies are needed, as well as strict standardized monitoring criteria to establish more reliable conclusions to assess the long-term effectiveness and safety of these methods. This meta-analysis assesses two treatment methods for CIN (cold cone and cryotherapy) compared to LEEP, including only randomized controlled studies to assess their efficacy and safety. It thus updates the evidence available to date. The main limitation of this review is that there are few randomized controlled studies, dating back more than 15 years and with short monitoring time. However, our systematic review addresses extensively the efficacy and safety outcomes with a specific analysis for each of them, thus providing a more refined analysis and better evidence for decision making in the therapeutic management of cervical intraepithelial neoplastic lesions.
