# Supplementary material for: Efficacy and safety of cryotherapy, cold cone or thermocoagulation compared to LEEP as a therapy for cervical intraepithelial neoplasia: Systematic review
Source: Rev Saude Publica. 2020 Mar 9;54:27. doi: 10.11606/s1518-8787.2020054001750 (PMC7063859; doi:10.11606/s1518-8787.2020054001750)
Supplement: Translation

# Translation: Eficacia y seguridad de crioterapia, cono frio o termo-coagulación en comparación con LEEP como terapia para la neoplasia intraepitelial cervical: Revisión sistemática.

**Authors:** Yamilée Hurtado-Roca, Naysha Becerra-Chauca, Magaly Malca

**Affiliations:** I Perú Instituto de Evaluación de Tecnologías en Salud e Investigación (IETSI), EsSalud. Lima, Perú; II Perú Hospital Rebagliati Martins, Servicio de Ginecología Oncológica, EsSalud. Lima, Perú

**Keywords:** Neoplasia Intraepitelial Cervical, terapia, Crioterapia, Conización, Electrocirugía, Neoplasias del Cuello Uterino, Revisión Sistemática

## Abstract

**OBJETIVOS:**

Evaluar la eficacia y seguridad del uso de crioterapia, cono frio o termo-coagulación en comparación con el procedimiento de escisión electroquirúrgica en asa (LEEP) para el manejo de neoplasias intraepiteliales cervicales.

**MÉTODOS:**

Revisión sistemática de ensayos controlados aleatorizados en mujeres con neoplasia intraepitelial cervical en tratamiento con crioterapia, cono frio, o termo coagulación y LEEP, para estimar su eficacia y seguridad. La búsqueda se realizó en MEDLINE/PUBMED, Registro Cochrane Central de Ensayos Controlados (CENTRAL) y Scopus, hasta setiembre de 2018.

**RESULTADOS:**

Se identificaron 72 estudios, ocho cumplieron los criterios de inclusión. Cono frio disminuyó el riesgo de enfermedad residual en comparación con LEEP (RR 0,54; IC del 95%, 0,30–0,96, p = 0,04). Crioterapia en comparación con LEEP incrementó el riesgo de recurrencia de enfermedad en un 86,0% (RR 1,86; IC del 95%, 1,16–2,97, p = 0,01) con un tiempo de seguimiento de seis a 24 meses, y de infecciones (RR, 1,17; IC del 95%, 1,08–1,28, p < 0,001); pero redujo el riesgo de sangrado menor en un 51,0% (RR 0,49; IC del 95%, 0,40–0,59, p ≤ 0,001).

**CONCLUSIONES:**

Cono frio reduce el riesgo de enfermedad residual. Sin embargo, la crioterapia reduce el riesgo de sangrado menor en las 24 horas posteriores al tratamiento e incrementa el riesgo de recurrencia de enfermedad y de infecciones.

## INTRODUCCION

El cáncer de cuello uterino es el cuarto tipo más común de cáncer y la cuarta causa de mortalidad en las mujeres a nivel mundial ^1^ . Sin embargo, el cáncer de cuello uterino se posiciona aún como la segunda causa de mortalidad y el segundo tipo de cáncer más común en la población femenina en los países de bajos y medianos ingresos ^1^ . La neoplasia intraepitelial cervical (NIC) es una lesión premaligna del cáncer de cuello uterino, histológicamente dividida como NIC1, NIC2 y NIC3. Tanto estas lesiones premalignas como el cáncer *in situ* son atribuidos al virus papiloma humano (VPH) ^2 , 3^ . De acuerdo con el reporte de la Agencia Internacional de Investigación del Cáncer (IARC) del 2018 ^4^ , en el Perú, los tipos oncogénicos 16 y 18 de VPH se encuentran en el 6,6% de pacientes con citología normal, en el 27,3% de NIC1, en el 53,1% de casos de NIC2-3 y en el 65,9% de cáncer *in situ* . El diagnóstico y manejo precoz de estas lesiones premalignas contribuyen a disminuir el avance natural de estas lesiones hasta convertirse en un cáncer cérvico uterino. El seguimiento de las lesiones histológicas NIC2 y NIC3 se convierte en una tarea fundamental en salud pública dado que el 31,0% de estas evolucionan a cáncer en los 30 años siguientes ^5^ . Una intervención terapéutica oportuna y adecuada puede disminuir este riesgo. Algunos autores han evidenciado en cohortes de seguimiento de 10–20 años que la tasa postratamiento de las lesiones premalignas disminuye en más del 30,0% durante los 10 primeros años ^6 , 7^ .

Para el tratamiento de las lesiones premalignas cervicouterinas, tanto los métodos ablativos (crioterapia cervical, ablación con láser) como los métodos de escisión (procedimiento de escisión electroquirúrgica en asa – Loop Electrosurgical Excision Procedure (LEEP), cono frío) pueden ser eficaces. Según lo recomendado por las pautas de la Organización Mundial de la Salud en la guía clínica publicada el 2015 ^8^ , en las pacientes con lesiones NIC2+, recomienda el tratamiento con crioterapia. Si no es elegible para esta terapia ablativa, se recomienda el uso del LEEP. Sin embargo, los procedimientos de escisión y ablación podrían asociarse con resultados adversos. Por lo tanto, el objetivo de esta revisión sistemática y meta análisis fue evaluar la eficacia y la seguridad del tratamiento de NIC con crioterapia, cono frio o termo coagulación en comparación con LEEP.

## MÉTODOS

Se siguieron las pautas de PRISMA (Preferred Reporting Items for Systematic Reviews and Meta-Analyses) para el desarrollo y reporte de esta revisión sistemática ^9^ .

Se consideraron elegibles ensayos controlados aleatorizados (ECA) (dado que el objetivo fue evaluar eficacia y seguridad) que cumplieron los siguientes criterios: población de estudio mujeres mayores de 18 años con diagnóstico de neoplasia intraepitelial cervical e intervenciones de tratamiento con crioterapia, cono frio, o termo coagulación y LEEP como comparador. Se excluyeron estudios si las participantes fueron mujeres embarazadas, mujeres con infección por VIH (Virus de Inmunodeficiencia Humana), mujeres sintomáticas o con antecedente de tratamiento y seguimiento por cáncer de cuello uterino.

Las variables para evaluar eficacia fueron: enfermedad residual (en menos de seis meses), enfermedad recurrente (en más de seis meses) y márgenes positivos. Para valorar seguridad, se evaluaron: sangrado mayor (hospitalización o transfusión de sangre), sangrado menor (sangrado que no requiere hospitalización ni transfusión de sangre después de las 24 horas posteriores al tratamiento), mortalidad asociada al tratamiento, estenosis cervical, dolor en zona de tratamiento, infecciones relacionadas al procedimiento (requiere hospitalización o antibióticos) y daño de otros órganos o necesidad de otras cirugías.

Se exploraron las bases de datos MEDLINE/PUBMED, Registro Cochrane Central de Ensayos Controlados (CENTRAL) *The Cochrane Library* y Scopus, Se utilizaron términos “Medical Subject Headings (MeSH)” o equivalentes y los términos de palabras de texto. Se incluyeron artículos en idioma inglés y español. Se creó una estrategia de búsqueda preliminar para MEDLINE/PUBMED. Las demás búsquedas se adaptaron a las bases de datos individualmente ( Tabla 1 ), la búsqueda se realizó desde enero de 1993 hasta setiembre del 2018 (últimos 25 años). Adicionalmente, evaluamos las referencias bibliográficas de los artículos seleccionados para identificar otros artículos relacionados a nuestra revisión sistemática. Utilizamos una estrategia de búsqueda amplia con el objetivo de incrementar la sensibilidad e identificar una cantidad relevante de artículos relacionados a nuestra pregunta de investigación. Elaboramos la siguiente estrategia de búsqueda en MEDLINE:

**Tabla 1:** Características de los estudios incluidos (1993–2018).

Estudio	Intervención			Histología	Diseño de estudio	Periodo de estudio	País	Tiempo de seguimiento	Tipo de centro de estudio	Profesional que hizo el procedimiento	Desenlace
	Cono Frio	Crioterapia	LEEP								
Chirenje 2001	-	200	200	NIC 2-3	ECA	1997–1998	Zimbabue	12 meses	Centro de tamizaje	Ginecólogo	Recurrencia de enfermedad (después de 6 meses de tratamiento) y Enfermedad residual (dentro de 6 meses de tratamiento)
Mitchell 1998	-	139	130	NIC 1-3	ECA	1992–1994	Estados Unidos	24 meses	Centro especializado	Tres ginecólogos, dos médicos de familia y dos enfermeras practicantes	Recurrencia de enfermedad (después de 6 meses de tratamiento), enfermedad residual (dentro de 6 meses de tratamiento), estenosis cervical (que requiera dilatación), sangrado menor (que requiera visita al hospital), dolor secundario (que requiera medicación) e infecciones (que requieran tratamiento antibiótico).
Duggan 1999	89	-	91	NIC 1-3	ECA	1992–1994	Estados Unidos	12 meses	Hospital especializado en mujeres	Residentes de Ginecología, con supervisión directa del investigador	Recurrencia de enfermedad (NE), Enfermedad residual (NE), Sangrado menor (hasta 6 semanas post operatorias), Estenosis cervical (NE), Infecciones (NE).
Giacalone 1999	38	-	28	NIC 2-3	ECA	1997–1998	Francia	3 meses	Hospital universitario	Médico entrenado	Enfermedad residual (NE), Sangrado secundario (que requiera visita al hospital), márgenes positivos y estenosis cervical (imposibilidad de inserción de dilatador Heger Nº 3).
Girardi 1994	38	-	52	NIC 1-3	ECA	No especifica	Austria	10 meses	Universidad	No especifica	Recurrencia de enfermedad (hasta los 8 o 10 meses), sangrado menor (NE)
Mathevet 1994	37	-	36	NIC 1-3	ECA	1990–1992	Canadá	6 meses	Hospital general	Médicos entrenados	Enfermedad residual (NE), estenosis cervical (imposibilidad de inserción de dilatador Heger Nº 3), sangrado menor (NE)y márgenes positivos.
Mathevet 2003	37	-	36	NIC 1-3	ECA	1990–1992	Canadá	65 meses	Hospital general	Médicos entrenados	Enfermedad residual, estenosis cervical, sangrado menor y márgenes positivos.
Takac 1999	120	-	120	NIC 1-3	ECA	1993–1996	Eslovenia	3 meses	Hospital especializado	No especifica	Enfermedad residual (hasta los 3 meses), sangrado menor (NE).

LEEP (Loop Electrosurgical Excision Procedure): Escisión electroquirúrgica en asa

ECA: Ensayo clínico aleatorizado. NIC: Neoplasia intraepitelial cervical.

NE: No Especifica

#1 (“Uterine Cervical Neoplasms”[Mesh]) OR ((“Uterine Cervical”[tiab] OR cervix[tiab]) AND (cancer[tiab] OR tumor[tiab] OR neoplasm[tiab] OR carcinoma[tiab] OR malignancy[tiab]))

#2 “Loop Electrosurgical Excision Procedure”[tiab] OR “Large loop excision of the transformation zone”[tiab] OR “LEEP”[tiab] OR “LLETZ”[tiab]

#3 “cryotherapy”[MeSH Terms] OR cryotherapy[tiab]

#4 “cold coagulation”[tiab]

#5 “conization”[MeSH Terms] OR “conization”[tiab]

#6 “Randomized Controlled Trial” [pt] OR “Randomized Controlled Trial”[tiab]

#7 #3 OR #4 OR #5

#8 AND #2 AND #7

#9 #8 AND #6

Para las bases de datos Scopus, y The Cochrane Library se elaboró la estrategia de búsqueda con los términos: “Uterine Cervical Neoplasms”, “Loop Electrosurgical Excision Procedure” y “Randomized Controlled Trial”.

### Selección de estudios y extracción de datos

Dos revisores evaluaron independientemente la elegibilidad de los estudios según títulos y resúmenes (YHR y NBC); las discordancias fueron resueltas por un tercer revisor (JG). Se evaluó independientemente los títulos y resúmenes del total de referencias seleccionadas aplicando los objetivos y la pregunta de investigación (PICO). Se revisó manualmente todas las referencias de los textos completos seleccionados.

De acuerdo con los criterios de búsqueda en las distintas bases de datos preseleccionadas, las referencias de cada base que cumplieron con los criterios de búsqueda fueron exportadas al software Zotero. Se eliminaron los duplicados y la información requerida (descripción de la metodología, resultados y conclusiones) fue extraída de forma independiente. Para la extracción de los datos principales, se utilizó el programa Review Manager 5.3 (RevMan 5.3, Copenhagen, The Cochrane Collaboration) ^10^ .

Para valorar el riesgo de sesgo de cada uno de los estudios, se utilizó la herramienta propuesta por Higgins ^11^ , siguiendo la metodología de la Colaboración Cochrane. Esta valoración incluyó la evaluación de los dominios: generación de la secuencia, ocultación de la asignación, cegamiento, datos de resultado incompletos, notificación selectiva de los resultados y otras fuentes de sesgo.

Se utilizó la metodología GRADE (Grading of Recommendations Assessment, Development and Evaluation) ^12^ para evaluar la certeza de la evidencia de cada desenlace. Esta valoración incluyó el riesgo de sesgo, inconsistencia, imprecisión, evidencia indirecta y otras consideraciones ^13^ .

Se realizó la síntesis cuantitativa de los estudios elegibles para los resultados binarios utilizando un modelo de efectos fijos permitiendo estimar el RR agrupado y sus intervalos al 95% de confianza. del efecto de los tratamientos para NIC de estudios similares. Se hicieron análisis de sensibilidad con efectos aleatorios si los resultados presentaron heterogeneidad (I ^2^ > 50%). Se realizaron pruebas de heterogeneidad (utilizando la prueba de chi-cuadrado de la heterogeneidad y la prueba estadística I ^2^ ) para cada uno de los resultados. La heterogeneidad fue aceptada si I ^2^ > 50% y se seleccionó un p de 0,1 como punto de corte para el rechazo de la hipótesis nula de la homogeneidad del estudio ^14^ .

Este estudio no requirió de aprobación de un comité de ética debido a que no es una data identificable ni es data individual.

## RESULTADOS

Se identificaron 72 artículos científicos: 66 de los registros identificados mediante la búsqueda de bases de datos (Cochrane = 22, PubMed = 19 y Scopus = 25) y seis a través de registros adicionales identificados a través de otras fuentes. Luego de eliminar duplicados, quedaron 51 registros para lectura de resúmenes, eliminándose 42 artículos. Tras la aplicación de los criterios de selección y luego de la lectura a texto completo, se seleccionaron ocho artículos originales ^15^ ( Tabla 1 ). El artículo de Huang y cols. ^22^ fue excluido por no ser un ensayo controlado aleatorizado. El 57,0% de los estudios incluidos tuvieron una adecuada generación aleatoria de la secuencia y describieron de forma clara el método de ocultamiento de la asignación. El 29,0% realizaron un cegamiento de participantes y del personal, y cegamiento de la evaluación del evento. Cuatro estudios tuvieron datos incompletos a evaluar al finalizar el tratamiento lo que generaría un riesgo de sesgo por pérdida de seguimiento. Tres de los estudios no especificaron claramente los resultados a valorar, por lo que se consideró un alto riesgo de sesgo de reporte ( Figura 1 ). Pudo observarse una mejor calificación en la evaluación del riesgo de sesgo en los estudios de Duggan y cols. ^19^ , Giacalone y cols. ^15^ , y Mitchell y cols. ^17^ .

**Figura 1:** Resumen por Dominios de la Evaluación de Riesgos de Sesgos según la Herramienta Cochrane (1993–2018).

Seis de los estudios incluidos evaluaron el tratamiento de NIC con cono frio en comparación con LEEP ^15 , 16 , 19^ , dos estudios evaluaron el tratamiento con crioterapia en comparación con LEEP ^17 , 18^ . No se identificó ningún estudio que evaluara el tratamiento de NIC con termo coagulación en comparación con LEEP. En relación con la comparación entre Cono Frío versus LEEP, la certeza de la evidencia fue muy baja para todos los desenlaces ( Tabla 2 ). Para la comparación entre Crioterapia con LEEP, a excepción del desenlace de Sangrado Menor después de las primeras 24 horas post tratamiento que tuvo una certeza moderada, los demás desenlaces fueron de certeza baja o muy baja ( Tabla 2 ).

**Tabla 2:** Resumen de Evidencia GRADE (1993–2018)

Desenlace	Efectos Absolutos Anticipados		Efecto Relativo (95% IC)	Nº de participantes (Estudios )	Certeza de la Evidencia (GRADE)
	Riesgo con LEEP	Riesgo con Cono Frío			
Pregunta: ¿Debería usarse Cono frío vs. LEEP para tratamiento de Neoplasia Intraepitelial Cervical (NIC)? Bilbiografía: Girardi et al 1994; Mathevet et al, 1994; Duggan et al, 1999; Takac et al 1999; Giacalone et al, 1999; Mathevet et al, 2003					
Recurrencia de Enfermedad (seguimiento: rango 6 meses a 118 meses)	71 por 1.000	23 por 1.000 (6 a 81)	RR 0,32 (0,09 a 1,14)	287 (3 ECAs)	⨁◯◯◯ MUY BAJA ^a,b^
Enfermedad Residual: (seguimiento: hasta los 6 meses)	112 por 1.000	61 por 1.000 (34 a 108)	RR 0,54 (0,30 a 0,96)	529 (4 ECAs)	⨁◯◯◯ MUY BAJA ^c,d^
Márgenes Positivos	212 por 1.000	164 por 1.000 (115 a 232)	RR 0,77 (0,54 a 1,09)	553 (4 ECAs)	⨁◯◯◯ MUY BAJA ^c,d,e^
Sangrado Menor durante las primeras 24 horas post tratamiento	54 por 1.000	57 por 1.000 (27 a 119)	RR 1,05 (0,50 a 2,21)	469 (4 ECAs)	⨁◯◯◯ MUY BAJA ^c,f^
Sangrado Menor después de las primeras 24 horas post tratamiento	88 por 1.000	83 por 1.000 (36 a 187)	RR 0,94 (0,41 a 0,13)	247 (2 ECAs)	⨁◯◯◯ MUY BAJA ^c,f^
Estenosis Cervical (seguimiento: rango 3 meses a 24 meses)	66 por 1.000	70 por 1.000 (29 a 169)	RR 1,06 (0,44 a 2,58)	529 (4 ECAs)	⨁◯◯◯ MUY BAJA ^c,f^

**Desenlace**	**Efectos Absolutos Anticipados**		**Efecto Relativo (95% IC)**	**Nº de participantes (Estudios )**	**Certeza de la Evidencia (GRADE)**
	**Riesgo con LEEP**	**Riesgo con Crioterapia**			
Pregunta: ¿Debería usarse Crioterapia vs. LEEP para tratamiento de Neoplasia Intraepitelial Cervical (NIC)? Bilbiografía: Mitchell et al, 1998; Chirenje et al, 2001					
Recurrencia de Enfermedad (seguimiento: rango de 6 a 24 meses)	77 por 1.000	144 por 1.000 (90 a 229)	RR 1,86 (1,16 a 2,97)	598 (2 ECAs)	⨁◯◯◯ MUY BAJA ^a,b^
Enfermedad Residual: (seguimiento: hasta los 6 meses)	37 por 1.000	65 por 1.000 (31 a 133)	RR 1,75 (0,85 a 3,60)	596 (2 ECAs)	⨁◯◯◯ MUY BAJA ^a,b^
Sangrado Menor durante las primeras 24 horas post tratamiento	15 por 1.000	4 por 1.000 (1 a 25)	RR 0,27 (0,04 a 1,62)	669 (2 ECAs)	⨁◯◯◯ MUY BAJA ^c,d^
Sangrado Menor después de las primeras 24 horas post tratamiento	484 por 1.000	237 por 1.000 (194 a 286)	RR 0,49 (0,40 a 0,59)	625 (2 ECAs)	⨁⨁⨁◯ MODERADO ^c^
Estenosis Cervical: (seguimiento: hasta los 24 meses)	3 por 1.000	6 por 1.000 (1 a 68)	RR 1,87 (0,17 a 20,38)	596 (2 ECAs)	⨁◯◯◯ MUY BAJA ^a,d^
Dolor después de 24h post tratamiento	275 por 1.000	256 por 1.000 (204 a 322)	RR 0,93 (0,74 a 1,17)	625 (2 ECAs)	⨁⨁◯◯ BAJA e,f
Infecciones después de 24h post tratamiento	465 por 1.000	544 por 1.000 (502 a 595)	RR 1,17 (1,08 a 1,28)	625 (2 ECAs)	⨁◯◯◯ MUY BAJA ^a,b^

GRADE (Grados de Certeza de evidencia); Escisión electroquirúrgica en asa (LEEP: Loop Electrosurgical Excision Procedure)

Alta certeza: estamos muy seguros de que el verdadero efecto se asemeja al de la estimación del efecto

Certeza moderada: confiamos moderadamente en la estimación del efecto: es probable que el efecto real sea cercano a la estimación del efecto, pero existe la posibilidad de que sea sustancialmente diferente

Baja certeza: nuestra confianza en la estimación del efecto es limitada: el efecto real puede ser sustancialmente diferente de la estimación del efecto

Muy baja certeza: tenemos muy poca confianza en la estimación del efecto: es probable que el efecto real sea sustancialmente diferente de la estimación del efecto

a. Se decidió disminuir dos niveles por Alto riesgo de Sesgo de Desgaste (ambos estudios tuvieron más del 10% de pérdidas)

b. Se decidió disminuir un nivel en imprecisión por el amplio IC que atraviesa el límite de 1,25.

c. Se decidió disminuir un nivel por Incierto Riesgo de sesgo de detección (incertidumbre en el cegamiento de los evaluadores)

d. Se decidió disminuir dos niveles en imprecisión por el amplio IC que atraviesa los límites 0,75 y 1,25 y por la pequeña cantidad de eventos.

e. Se decidió disminuir un nivel por Incierto Riesgo de detección (cegamiento de evaluadores).

f. Se decidió disminuir un nivel en imprecisión por el amplio IC que atraviesa el límite de 0,75

Tres de los seis estudios incluidos evaluaron la recurrencia de enfermedad ^19 , 20 , 23^ . La prevalencia de recurrencia de enfermedad después del tratamiento con cono frio fue 2,0% y en las pacientes tratadas con LEEP, en 7,1%. El estudio de Girardi y cols. ^20^ no identificó ningún caso de recurrencia de enfermedad en ninguna de las pacientes tratadas con cono frio o LEEP. El metaanálisis de los estudios evidenció que no existe una diferencia riesgo estadísticamente significativa de recurrencia de enfermedad entre los pacientes en tratamiento de NIC con cono frio en comparación con LEEP (RR 0,32; IC del 95%, 0,09–1,14, p = 0,08). Los estudios no mostraron heterogeneidad significativa (P = 0,65 y I ^2^ = 0%) (Figura 2A).

**Figura 2:** Tratamiento de neoplasia intraepitelial cervical (NIC) con Cono frio versus Escisión electroquirúrgica en asa (LEEP: Loop Electrosurgical Excision Procedure).

Cuatro estudios evaluaron enfermedad residual ^15 , 16 , 19 , 21^ . Se reportó enfermedad residual en 6,1% de las pacientes tratadas con cono frio y en 11,2% de las tratadas con LEEP. El meta análisis de los cuatro estudios mostró que las pacientes tratadas con cono frio tienen menor probabilidad de presentar enfermedad residual en comparación de las tratadas con LEEP (RR 0,54; IC del 95%, 0,30–0,96, p = 0,04). Los estudios no mostraron heterogeneidad significativa (P = 0,88 y I ^2^ = 0%) (Figura 2B).

Cuatro estudios evaluaron márgenes positivos ^15 , 16 , 20 , 21^ . Se reportaron casos tanto con el uso de cono frio como con LEEP. En las pacientes tratadas con cono frio, se reportaron 16,1% de los casos y en las tratadas con LEEP se registraron 21,3% de los casos. El meta análisis de los cuatro estudios mostró que no existe un riesgo estadísticamente significativo de presentar márgenes positivos en pacientes en tratamiento de NIC con cono frio en comparación con LEEP (RR 0,77; IC del 95%, 0,54–1,09, p = 0,14) (Figura 2C). Los estudios mostraron moderada heterogeneidad (P = 0,09 y I ^2^ = 54%), por lo que se realizó un análisis de sensibilidad aplicando efectos aleatorios, y no hubo cambios significativos en el resultado (RR 0,77; IC del 95%, 0,44–1,35, p = 0,36).

Cuatro estudios incluidos evaluaron este desenlace ^15 , 16 , 20 , 21^ . La prevalencia de sangrado menor en las pacientes tratadas con cono frio fue 5,7% y en las tratadas con LEEP de 5,4%. Después de realizar el meta análisis de los cinco estudios, el riesgo de presentar sangrado menor durante las primeras 24 horas posteriores al tratamiento en las pacientes tratadas con cono frio *versus* LEEP no fue estadísticamente significativo (RR 1,05; IC del 95%, 0,50–2,21, p = 0,90). No se identificó heterogeneidad significativa de los estudios incluidos (P = 0,92 y I ^2^ = 0%) (Figura 2D).

Dos estudios evaluaron sangrado menor después de las primeras 24 post tratamiento ^16 , 19^ . El 8,2% de las pacientes tratadas con cono frio reportaron eventos de sangrado menor después de las 24 horas del tratamiento, la prevalencia de este evento en las pacientes tratadas con LEEP fue de 8,8%. Después de realizar el meta análisis de los estudios, el riesgo de presentar sangrado menor después de las primeras 24 horas posteriores al tratamiento en las pacientes tratadas con cono frio versus LEEP no fue estadísticamente significativo (RR, 0,94; IC del 95%, 0,41–2,13, p = 0,88). No se identificó heterogeneidad significativa de los estudios incluidos (P = 0,97 y I ^2^ = 0%) (Figura 2E).

Tres estudios incluidos evaluaron estenosis cervical ^15 , 16 , 19^ . La prevalencia después del tratamiento con cono frio fue de 6,9% y de 6,5% en las pacientes tratadas con LEEP. El meta análisis mostró mayor riesgo de estenosis cervical en mujeres tratadas con cono frio versus LEEP, sin embargo, este resultado no fue estadísticamente significativo (RR 1,06; IC del 95%, 0,44–2,58, p = 0,89). Giacalone y cols. ^15^ no identificaron casos de estenosis cervical en ninguna de las pacientes tratadas con cono frio o LEEP. Los estudios mostraron una baja heterogeneidad (P = 0,30 y I ^2^ = 5%) (Figura 2F).

Dugan y cols ^19^ reportaron un caso de infección en el sitio de operación en cada uno de los grupos. Los seis estudios evaluados no reportaron sangrado mayor ni dolor secundario al tratamiento con cono frio ni LEEP.

Para la comparación de tratamientos de NIC con Crioterapia versus LEEP, identificamos dos estudios ^17 , 18^ . Ambos estudios reportaron 14,7% de recurrencia de enfermedad en pacientes tratadas con crioterapia y 7,7% de las tratadas con LEEP. El meta análisis mostró mayor riesgo de recurrencia de enfermedad en mujeres tratadas con crioterapia en comparación con las tratadas con LEEP (RR 1,86; IC del 95%, 1,16–2,97, p = 0,01) (Figura 3A). Los estudios mostraron una moderada heterogeneidad (P = 0,14 y I ^2^ = 53%) por lo que se realizó un análisis de sensibilidad aplicando efectos aleatorios, y no hubo cambios significativos en el resultado (RR 1,86; IC del 95%, 1,16–2,97, p = 0,01).

**Figura 3:** Tratamiento de neoplasia intraepitelial cervical (NIC) con Crioterapia versus Escisión electroquirúrgica en asa (LEEP: Loop Electrosurgical Excision Procedure).

Para el tratamiento con crioterapia versus LEEP se reportaron casos de enfermedad residual en los dos estudios seleccionados. El 6,3% de las pacientes tratadas con crioterapia y el 3,7% de las tratadas con LEEP presentaron este evento. El meta análisis mostró mayor riesgo de enfermedad residual en mujeres tratadas con crioterapia en comparación con las tratadas con LEEP, sin embargo, fue estadísticamente no significativo (RR 1,75; IC del 95%, 0,85–3,60, p = 0,13). Los estudios no mostraron heterogeneidad (P = 0,91 y I ^2^ = 0%) (Figura 3B). La prevalencia de sangrado menor en las pacientes tratadas con crioterapia fue 0,3% y de 1,5% en las tratadas con LEEP. Después de realizar el meta análisis de los estudios, el riesgo de presentar sangrado menor durante las primeras 24 horas posteriores al tratamiento en las pacientes tratadas con crioterapia versus LEEP no fue estadísticamente significativo (RR 0,27; IC del 95%, 0,04–1,62, p = 0,15). No se identificó heterogeneidad significativa de los estudios incluidos (P = 0,91 y I ^2^ = 0%) (Figura 3C).

El 22,0% de las pacientes tratadas con crioterapia reportaron eventos de sangrado menor después de las 24 horas del tratamiento, la prevalencia de este evento en las pacientes tratadas con LEEP fue de 48,4%. Después de realizar el meta análisis, las pacientes tratadas con crioterapia tuvieron menor riesgo de sangrado después de las 24 horas posteriores al tratamiento en comparación con las tratadas con LEEP (RR 0,49; IC del 95%, 0,40–0,59, p < 0,001). No se identificó heterogeneidad significativa de los estudios incluidos (P = 0,17 y I ^2^ = 46%) (Figura 3D).

Chirenje y cols. ^18^ no reportaron casos de estenosis cervical en ninguno de los grupos de tratamiento. Sin embargo, Mitchell y cols. ^17^ reportaron dos casos en las pacientes tratadas con crioterapia (139) y un caso en el grupo de pacientes tratadas con LEEP (130), con un riesgo no significativo de presentar estenosis cervical si son tratadas con crioterapia en comparación con LEEP (RR 1,87; IC del 95%, 0,17–20,38, p = 0,61) (Figura 3E).

Los dos estudios identificados reportaron casos de dolor secundario al tratamiento. La prevalencia de dolor en las pacientes tratadas con crioterapia fue 23,9% y de 27,5% en las tratadas con LEEP. El meta análisis no mostró un riesgo significativo de presentar dolor secundario al tratamiento con crioterapia en comparación con LEEP (RR 0,93; IC del 95%, 0,74–1,17, p = 0,54). Los estudios no mostraron heterogeneidad (P = 0,50 y I ^2^ = 0%).

Ambos estudios reportaron casos de infecciones post tratamiento. La prevalencia en el grupo tratado con crioterapia fue de 51,1% y en el grupo tratadas con LEEP de 46,5%. Después del meta análisis de los estudios, las pacientes tratadas con crioterapia tuvieron más riesgo de hacer infecciones en la herida operatoria en comparación con las tratadas con LEEP (RR 1,17; IC del 95%, 1,08–1,28, p < 0,001). Los estudios no mostraron heterogeneidad (P = 0,87 y I ^2^ = 0%) (Figura 3F).

## DISCUSIÓN

En esta revisión sistemática, identificamos escasos ensayos controlados aleatorizados que evaluaron el tratamiento de NIC con crioterapia o cono frío en comparación con LEEP (dos y seis estudios, respectivamente). Los estudios tuvieron moderada calidad metodológica. No se identificó una heterogeneidad significativa entre los estudios evaluados para cada uno de los desenlaces de eficacia y seguridad, excepto para márgenes positivos en los estudios que comparaban cono frio versus LEEP y para recurrencia de enfermedad para los estudios que comparaban crioterapia con LEEP. El meta análisis de los datos de los estudios incluidos evidenció que el uso cono frio disminuye el riesgo de enfermedad residual en comparación con LEEP. Mientras que el uso de crioterapia incrementa el riesgo de recurrencia de enfermedad y de infecciones; sin embargo, reduce el riesgo de sangrado menor en comparación con el tratamiento con LEEP. En discrepancia con el estudio de Jiang y cols. ^24^ , evidenciamos que existe un menor riesgo de enfermedad residual en las pacientes tratadas con cono frio en comparación con LEEP. Este hallazgo puede sugerir que la técnica usada podría relacionarse con la profundidad del tejido extraído, revelado en la positividad de los márgenes, que Jin y cols. ^25^ en su meta-análisis, señala como un factor pronóstico de recurrencia y/o enfermedad residual. La Revisión sistemática de El-Nashar y cols. ^26^ evidenció menor probabilidad de sangrado post operatorio y estenosis cervical en el manejo de NIC con LEEP. Sus resultados muestran un incremento de hasta dos veces en la incidencia de recurrencia de enfermedad en las pacientes tratadas con LEEP en comparación con las que fueron manejadas con cono frio. Estos resultados están en discrepancia con nuestros hallazgos. No hemos identificado diferencias en el manejo de lesiones premalignas con estos dos métodos para los mismos desenlaces. Esta discrepancia puede ser atribuida a la diferencia de tipos de estudios incluidos en ambas revisiones. El estudio de El-Nashar y cols. ^26^ incluyó tanto ensayos controlados aleatorizados como estudios observacionales y no realizaron análisis estratificados para cada uno de estos tipos de estudios. Dado que el objetivo de nuestro estudio fue evaluar eficacia y seguridad, nuestra revisión sistemática se restringió a la evaluación de ECA.

Nuestro estudio evaluó la eficacia y seguridad del manejo de NIC con crioterapia en comparación con LEEP. Una revisión sistemática previa de ensayos controlados sugiere que la tasa de recurrencia de enfermedad (≥ 6 meses post tratamiento) puede ser menor después del manejo de NIC con LEEP en comparación con crioterapia (RR 0,32; IC 95% 0,13–0,78) ^27^ . Nuestra revisión incluye además el estudio de Mitchell y cols. ^17^ encontrando una tasa de recurrencia de enfermedad incrementada casi dos veces con el uso de crioterapia en comparación con LEEP. Por lo tanto, nuestro estudio es consistente con la evidencia de mayor riesgo de recurrencia de enfermedad con el uso de crioterapia en comparación con LEEP. Del mismo modo, D’Alessandro y cols. ^28^ han publicado una revisión sistemática evaluando el manejo de NIC con LEEP y crioterapia. Los resultados de este estudio evidencian un menor riesgo de recurrencia de enfermedad en pacientes tratadas con LEEP en comparación con las tratadas con crioterapia (RR 0,87; IC 95% 0,76–0,99). Este hallazgo es similar al de nuestra revisión en el que evidenciamos que la eficacia del uso de LEEP es mayor en relación con crioterapia, dado que nosotros identificamos un mayor riesgo de recurrencia de enfermedad con el uso de crioterapia. Nuestro estudio no incluyó el estudio de Smith y cols. ^29^ ya que ellos evaluaron pacientes con VIH, ni el estudio de Singh y cols. ^30^ al ser un estudio de tipo observacional. Es fundamental destacar el contexto en el que los dos estudios incluidos realizaron los tratamientos: los profesionales fueron personal sanitario especialmente entrenado en el uso de ambos métodos y los centros de atención fueron unidades especializadas que podría incrementar las probabilidades de éxito de los procedimientos. Estos son puntos destacables a tener en cuenta al considerar su implementación. El uso de cono frío sería la mejor opción en comparación con LEEP en paciente con NIC, en términos de enfermedad residual. Por otro lado, el uso de LEEP sería la mejor opción en comparación con la crioterapia en términos de recurrencia e infección post operatoria. Sin embargo, esta evidencia es de muy baja certeza, según la metodología GRADE. El verdadero efecto puede ser sustancialmente distinto. Se requieren más estudios controlados aleatorizados, así como criterios estrictos de seguimiento estandarizados para establecer conclusiones más fidedignas que permitan valorar la eficacia y seguridad de estos métodos a largo plazo. Este meta análisis evalúa dos métodos de tratamiento de NIC (cono frio y crioterapia) en comparación con LEEP, incluyendo sólo ensayos controlados aleatorizados que permitan evaluar su eficacia y seguridad. Actualiza de este modo la evidencia disponible hasta la fecha. La principal limitación de esta revisión es que existen escasos ensayos controlados aleatorizados, datan de hace más de 15 años y tienen tiempos de seguimiento cortos. Sin embargo, nuestra revisión sistemática aborda ampliamente los desenlaces de eficacia y seguridad con un análisis específico para cada uno de ellos, aportando de este modo un análisis más fino y una mejor evidencia para la toma de decisiones en el manejo terapéutico de lesiones neoplásicas intraepiteliales cervicales.
